# Single‐layer versus double‐layer suture during laparoscopic partial nephrectomy for renal tumours in close proximity to the collecting system or renal sinus: A propensity‐matched analysis

**DOI:** 10.1002/bco2.70256

**Published:** 2026-08-02

**Authors:** Xiaofeng Xu, Xiuquan Shi, Xiumin Zhou, Yuhao Chen, Zhe Liu, Changjie Shi, Wen Cheng, Jingping Ge, Xuejun Shang

**Affiliations:** ^1^ Department of Urology First Affiliated Hospital of Soochow University Suzhou China; ^2^ Department of Urology Jinling Hospital of Nanjing University Nanjing China; ^3^ Department of Oncology First Affiliated Hospital of Soochow University Suzhou China

**Keywords:** laparoscopic partial nephrectomy, postoperative complications, single‐layer suture

## Abstract

**Objectives:**

To compares perioperative outcomes of single‐layer suture (SLS) and double‐layer suture (DLS) technique for laparoscopic partial nephrectomy (LPN) in patients with renal tumours in close proximity to the collecting system or renal sinus.

**Patients and Methods:**

A total of 178 patients with renal tumours adjacent to the collecting system or renal sinus were identified from the databases of our institution who underwent LPN between November 2020 and December 2023. SLS (*n* = 79) or DLS (*n* = 99) technique was used for renal defect suture. Propensity score matching was performed to minimize selection bias. Propensity scores were calculated for age, sex, body mass index, tumour diameter, renal nephrometry score, history of diabetes or hypertension, preoperative haemoglobin and preoperative estimated glomerular filtration rate (eGFR). Perioperative data were analysed.

**Results:**

In all, 76 patients could be matched in each group. Compared with DLS, SLS significantly reduced the warm ischaemia time (22.45 ± 7.75 vs. 28.87 ± 7.77 min, *p* < 0.001). There was no difference between the two groups for postoperative complications including transfusion‐treated blood loss (1.3% vs. 1.3%, *p* = 1.000). No grade III‐V complications occurred. Moreover, no significant difference was found in postoperative haemoglobin decline between the two groups (1.27 ± 1.20 vs. 1.40 ± 1.20 g/dL, *p* = 0.508).

**Conclusion:**

SLS during LPN offers effective renorrhaphy, reducing the warm ischaemia time without increasing the risk of complications.

## 1 INTRODUCTION

During partial nephrectomy, reconstructive techniques are the major determinants of ultimate renal function because they are strongly correlated with warm ischaemia time (WIT) and significantly affect the amount of vascularized parenchyma preserved.^1^, ^2^, ^3^, ^4^, ^5^

Currently, the ideal renorrhaphy technique remains debatable. Classically, double‐layer suture (DLS) is used for parenchymal renorrhaphy during laparoscopic partial nephrectomy (LPN). However, renorrhaphy sutures are time consuming and can compress the renal parenchyma, and more sutures will result in greater impairment of the postoperative renal function.^3^, ^6^, ^7^ Accordingly, a few surgeons use single‐layer suture (SLS) to treat renal defects.^7^, ^8^, ^9^, ^10^, ^11^ The application of SLS is believed to help shorten the WIT and may be associated with improved postoperative renal function.^6^, ^7^, ^10^, ^11^ However, most of these studies did not classify tumours into superficial or deep growth subgroups according to their growth depth. Therefore, it remains uncertain whether the SLS technique is safe for deep renal defect.

SLS and DLS techniques have been performed contemporaneously during LPN at our institution over the past years. In this study, we retrospectively analysed the perioperative outcomes of these techniques. To ensure the universal applicability of the results, the cases with the renal tumours in close proximity to the collecting system and renal sinus were selected. The intraparenchymal depth of tumour invasion was graded according to the N score of the RENAL nephrometry scoring system.^12^ Renal tumours in close proximity to the collecting system or renal sinus in this study refer to those near the collecting system or renal sinus ≤4 mm, with an *N* score of 3.

## 2 PATIENTS AND METHODS

### 2.1 Study design and data collection

**FIGURE 1:**
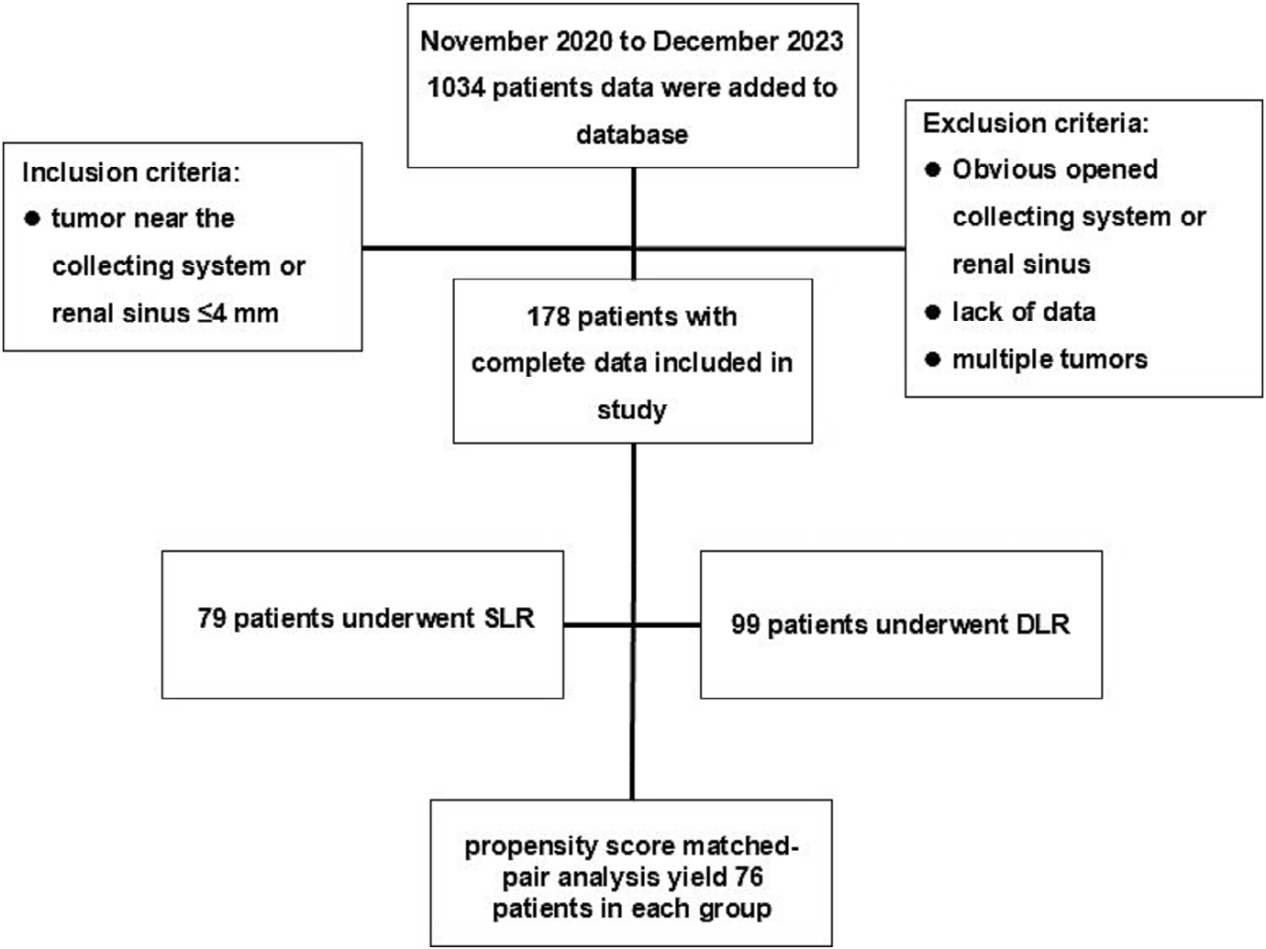
Flowchart showing the patient enrolment process.

This retrospective cohort study was approved by the Institutional Review Board of Jinling Hospital. After obtaining informed consent, we retrospectively collected data from consecutive patients who underwent LPN at our institution between November 2020 and December 2023. Inclusion criteria were as follows: CT or MRI suggested that the lesion was near the collecting system or renal sinus ≤4 mm. Cases whose collecting system had been obviously opened or renal sinus had been widely opened during operation or with missing preoperative images or perioperative data, or multiple tumours were excluded. Of 1034 patients who underwent LPN during the study period, 178 patients met the inclusion criteria and were enrolled. Of these, 79 underwent SLS technique for renorrhaphy of renal defects and 99 underwent the DLS technique. To minimize selection bias and confounding factors, propensity score matched‐pair analysis was performed between the SLS and DLS groups. Figure 1 shows the study flow chart. The perioperative factors analysed included WIT, estimated blood loss (EBL), postoperative haemoglobin decline, postoperative complications, length of postoperative hospital stay and length of follow‐up. The anatomical characteristics of the tumours were assessed preoperatively using CT or MRI. Patient performance status was evaluated using the American Society of Anesthesiologists classification of physical status. The estimated glomerular filtration rate (eGFR) from baseline were assessed using the CKD‐EPI Creatinine Equation (2021).^13^ Postoperative complications were classified into major (III–V) and minor (I–II) using the Clavien–Dindo grading system.^14^ Follow‐up data were obtained by reviewing patient charts or via telephone calls. Propensity score matching (PSM) was conducted. Preoperative and perioperative results were analysed and compared between the groups.

### 2.2 Surgical technique

All surgeries were performed by surgeons experienced in LPN. Three surgeons performed SLS technique and four surgeons performed DLS technique. All procedures were performed using a retroperitoneal approach. After the induction of general anaesthesia, the patient was placed in a full‐flank position. The level of the kidney bridge was elevated. The operation table was flexed to increase the distance between the ribs and iliac crest. Three ports were used in the procedure, with an additional port added if needed. Gerota's fascia was incised longitudinally. The kidneys were then mobilized. The renal artery was isolated and clamped with a bulldog clamp. The tumour was excised with a parenchyma margin using cold scissors. No electrocautery and haemostatic agents were used for haemostasis on the resection defect. Subsequently, a renorrhaphy was performed. A 20‐cm unidirectional barbed suture (2‐0 V‐Loc™ 180 absorbable suture; Covidien, Mansfield, MA, USA) with a knot and a Hem‐o‐lok clip at the tail end was used. In the SLS technique, closure of the entire tumour bed is performed using only one layer of running sutures. The needle was passed through the renal capsule and the whole thickness of the renal parenchyma, while deep sutures through the floor of the defect were avoided. In DLS, the resection bed is repaired using internal running sutures. Subsequently, an outer‐layer renorrhaphy was performed using running sutures. No Hem‐o‐lok clip was used to secure each stitch at this step. After the final tissue stitch, the thread was secured outside the renal capsule by using a Hem‐o‐lok clip. The bulldog clamp was removed after renorrhaphy was completed. Finally, the kidney was inspected, and haemostasis was confirmed.

### 2.3 Statistical analysis

Statistical analyses were performed using R (Version 4.4.2). PSM was performed using a 1:1 nearest neighbour matching algorithm without replacement, with a calliper of 0.2. Propensity scores were estimated via multivariate logistic regression incorporating the following covariates: age, sex, body mass index (BMI), tumour diameter, RENAL score, history of diabetes or hypertension, preoperative haemoglobin and eGFR. Covariate balance between the matched groups was assessed using standardized mean differences (SMDs). Continuous variables are presented as median (interquartile range) or mean ± standard deviation. Independent samples Student's *t*‐test (for normally distributed continuous variables), Mann–Whitney *U* test (for non‐normally distributed continuous variables) and chi‐square analysis (for categorical variables) were used to compare perioperative data, with statistical significance set at *p* < 0.05.

## 3 RESULTS

Table 1 presents a comparison of preoperative information before PSM in the SLS and DLS groups. After using 1:1 PSM, 76 patients per group were matched. The two groups showed a well balance of preoperative demographic and tumour parameters (Table 2).

**TABLE 1:** Prematching comparison of preoperative data between the two groups of patients.

Variables	SLS (*n* = 79)	DLS (*n* = 99)	*p* value	SMD
Age (years), mean ± SD	55.08 ± 11.17	55.11 ± 11.42	0.984	0.003
Sex (male), *n* (%)	55 (69.6)	67 (67.7)	0.908	0.019
BMI (kg/m^2^), mean ± SD	24.86 ± 2.66	25.31 ± 3.57	0.351	0.143
DM, *n* (%)	16 (20.3)	9 (9.1)	0.056	0.112
HTN, *n* (%)	30 (38.0)	34 (34.3)	0.731	0.036
ASA score, *n* (%)	2 (2–2)	2 (2–2)	>0.999	0.080
Tumour size (mm), mean ± SD	33.42 ± 9.08	34.87 ± 8.92	0.286	0.161
Right‐side tumour, *n* (%)	42 (53.2)	51 (51.5)	0.946	0.016
RENAL nephrometry score, median (IQR)	8 (7–9)	8 (7–9)	>0.999	0.225
RENAL complexity, *n* (%)			0.786	0.106
Low (4–6)	3 (3.8)	4 (4.0)		
Intermediate (7–9)	73 (92.4)	89 (89.9)		
High (10–12)	3 (3.8)	6 (6.1)		
Tumour location, *n* (%)			0.209	0.217
Upper pole	21 (26.6)	31 (31.3)		
Mid pole	24 (30.4)	38 (38.4)		
Lower pole	34 (43.0)	30 (30.3)		
Preop haemoglobin (g/dL), mean ± SD	13.66 ± 1.44	13.63 ± 1.62	0.909	0.017
Preop creatinine (mg/dL), mean ± SD	0.93 ± 0.24	0.96 ± 0.31	0.471	0.111
Preop eGFR (mL/min), median (IQR)	101.47 (93.57–109.32)	100.59 (95.59–108.22)	>0.999	0.093

Abbreviations: ASA, American Society of Anesthesiologists; BMI, body mass index; DLS, double‐layer suture; DM, diabetes mellitus; eGFR, estimated glomerular filtration rate; HTN, hypertension; IQR, interquartile range; RENAL, radius, endophytic/exophytic properties, nearness of tumour to collecting system or sinus, anterior/posterior location relative to polar lines; SD, standard deviation; SLS, single‐layer suture; SMD, standardized mean difference.

**TABLE 2:** Postmatching comparison of preoperative data between the two groups of patients.

Variables	SLS (*n* = 76)	DLS (*n* = 76)	*p* value	SMD
Age (years), mean ± SD	55.01 ± 11.35	56.03 ± 10.79	0.574	0.011
Sex (male), *n* (%)	52 (68.4)	52 (68.4)	1.000	0.031
BMI (kg/m^2^), mean ± SD	24.83 ± 2.69	24.56 ± 2.91	0.549	0.063
DM, *n* (%)	15 (19.7)	8 (10.5)	0.174	0.047
HTN, *n* (%)	27 (35.5)	29 (38.2)	0.866	0.016
ASA score, median (IQR)	2 (2–2)	2 (2–2)	>0.999	0.050
Tumour size (mm), mean ± SD	33.47 ± 9.23	33.59 ± 7.94	0.933	0.007
Right‐side tumour, *n* (%)	40 (52.6)	36 (47.4)	0.626	0.047
RENAL nephrometry score, median (IQR)	8 (7–9)	8 (7–9)	>0.999	0.036
RENAL complexity, *n* (%)			0.842	0.095
Low (4–6)	3 (3.9)	4 (5.3)		
Intermediate (7–9)	70 (92.1)	70 (92.1)		
High (10–12)	3 (3.9)	2 (2.6)		
Tumour location, *n* (%)			0.236	0.097
Upper pole	21 (27.6)	25 (32.9)		
Mid pole	23 (30.3)	29 (38.2)		
Lower pole	32 (42.1)	22 (28.9)		
Preop haemoglobin (g/dL), mean ± SD	13.66 ± 1.46	13.49 ± 1.61	0.477	0.060
Preop creatinine (mg/dL), mean ± SD	0.92 ± 0.24	0.99 ± 0.33	0.178	0.022
Preop eGFR (mL/min), median (IQR)	102.11 (95.80–109.60)	100.97 (91.55–108.16)	>0.999	0.015

Abbreviations: ASA, American Society of Anesthesiologists; BMI, body mass index; DLS, double‐layer suture; DM, diabetes mellitus; eGFR, estimated glomerular filtration rate; HTN, hypertension; IQR, interquartile range; RENAL, radius, endophytic/exophytic properties, nearness of tumour to collecting system or sinus, anterior/posterior location relative to polar lines; SD, standard deviation; SLS, single‐layer suture; SMD, standardized mean difference.

Table 3 provides a comparison of the intraoperative and postoperative parameters between two groups. The WIT was shorter in the SLS group (22.45 ± 7.75 vs. 28.87 ± 7.77 min, *p* < 0.001). The intraoperative EBL and intraoperative blood transfusion show no significant difference between the groups. No conversion to radical nephrectomy or open surgery was performed. There was no significant difference in the decline of haemoglobin between the SLS and DLS groups postoperatively (1.27 ± 1.20 vs. 1.40 ± 1.20 g/dL, *p* = 0.508). No margin positivity was observed in either group. The median follow‐up period in SLS and DLS group were 21.5 and 13 months, respectively. Disease‐specific mortality was not observed during follow‐up.

**TABLE 3:** Postmatching comparison of intraoperative and postoperative results between the two groups of patients.

Variables	SLS (*n* = 76)	DLS (*n* = 76)	*p* value
Intraoperative			
WIT (min), mean ± SD	22.45 ± 7.75	28.87 ± 7.77	<0.001
EBL (mL), mean ± SD	37.01 ± 99.75	47.76 ± 99.00	0.506
Intraoperative transfusion, *n* (%)	0	2 (2.6)	0.477
Conversion to radical nephrectomy, *n* (%)	0	0	
Postoperative			
Overall complications, *n* (%)	1 (1.3)	1 (1.3)	1.000
Clavien–Dindo grades I–II			
Blood loss treated with transfusion, *n* (%)	1 (1.3)	1 (1.3)	1.000
Clavien–Dindo grades III–V			
Blood loss treated with superselective embolization, *n* (%)	0	0	
Urine leak, *n* (%)	0	0	
Readmission, *n* (%)	0	0	
Postoperative hospital stay (days), median (IQR)	4.50 (3.00–6.00)	6.00 (4.75–7.00)	>0.999
Decrease of haemoglobin (g/dL), mean ± SD	1.27 ± 1.20	1.40 ± 1.20	0.508
Postoperative eGFR (mL/min), mean ± SD	91.42 ± 18.45	85.35 ± 22.34	0.070
% eGFR loss, mean ± SD	8.56 ± 13.03	11.38 ± 12.81	0.181
Positive surgical margins, *n* (%)	0	0	
Histology, *n* (%)			0.193
Malignant lesion			
RCC subtype			
Clear cell	48 (63.2)	60 (78.9)	
Papillary	7 (9.2)	4 (5.3)	
Chromophobe	3 (3.9)	3 (3.9)	
MTSCC	0	1 (1.3)	
Unclassified	1 (1.3)	0	
Benign	17 (22.4)	8 (10.5)	
RCC pathological stage, *n* (%)			0.126
PT1a	60 (78.9)	67 (88.2)	
PT1b	16 (21.1)	9 (11.8)	
Duration of follow‐up (months), median (IQR)	21.50 (10.00–38.00)	13.00 (7.00–27.25)	>0.999

Abbreviations: DLS, double‐layer suture; EBL, estimated blood loss; eGFR, estimated glomerular filtration rate; IQR, interquartile range; MTSCC, mucinous tubular and spindle cell carcinoma; SD, standard deviation; SLS, single‐layer suture; WIT, warm ischaemia time.

The postoperative complications were assessed. In total, two patients required blood transfusions for haemorrhage (one in the SLS group and one in the DLS group). No statistically significant differences were observed between groups (*p* = 1.000). No urine leak was observed in either group. Grade III‐V complications were not observed.

## 4 DISCUSSION

During nephron‐sparing surgery, renorrhaphy techniques are the most important factors, not only for WIT but also for preserving the amount of vascularized parenchyma.^1^, ^2^, ^3^, ^4^ However, no urological guidelines have provided recommendations for the optimal renorrhaphy technique.^15^, ^16^

The traditional DLS technique has been used for many years in minimally invasive partial nephrectomy (MIPN). It has been considered necessary to prevent postoperative bleeding and urine leakage. However, this conjecture has not yet been proven using data.

Despite achieving haemostasis and preventing urinary fistulas, renorrhaphy sutures can compress the renal parenchyma and decrease the renal blood flow. Blood flow occlusion after SLS is mainly localized to the resection site, whereas DLS can lead to renal tissue ischaemia remote from the tumour location.^7^ DLS can damage more renal vessels and renal parenchyma, leading to more postoperative loss of renal function.^6^, ^7^, ^10^, ^11^, ^17^, ^18^ In addition, blind sutures through the deep portions of the kidney have a higher risk of involving collecting system and developing renal artery pseudoaneurysms that would increase the complications rate.^1^, ^9^

Therefore, a few surgeons have gradually questioned whether DLS of renal defects is indispensable for reducing complications. They began using the SLS technique (cortical or medullary layer‐only renorrhaphy) to close renal parenchymal defects.^7^, ^8^, ^9^, ^10^, ^11^ In addition, sutureless technique was also used to suture the parenchymal breach.^19^

Bahler et al. compared 38 base‐layer‐only renorrhaphy patients with 118 double‐layer renorrhaphy patients and found similar bleeding complications and urine leaks between the two groups. A limitation of this study is that all base‐layer‐only renorrhaphy procedures were performed using an open approach, whereas the surgical procedures of the double‐layer renorrhaphy group comprised open and robotic approaches.^7^ In another study, Bahler et al. reported a medullary layer‐only renorrhaphy technique for robot‐assisted partial nephrectomy (RAPN). A running base‐layer suture was performed to close the collecting system and base layer; however, cortical renorrhaphy was omitted. No urine leakage or bleeding complications were observed in the 15 patients.^11^

Another study compared 40 inner‐layer‐only renorrhaphy cases to 106 double‐layer renorrhaphy cases during RAPN. Inner‐layer renorrhaphy was performed to close the collecting system and achieve haemostasis. Haemostatic agents were applied to the base layer. No significant differences were observed in postoperative complications between the two groups.^8^

Additionally, Bylund et al. reported that only the renal cortex was re‐approximated using bolstering sutures in 104 patients. No attempt has been made to close the collecting system even when it has been clearly entered. They found that their technique had similar urine leakage and transfusion rates to those of other series. However, their series exclusively involved hand‐assisted LPN instead of a purely intracorporeal technique, and a fibrin glue patch was placed within the surgical defect before bolstering sutures.^9^ Williams et al. also omitted inner‐layer renorrhaphy during RAPN. Cortical renorrhaphy was performed in 26 patients using the sliding‐clip technique without altering the complications, hospital stay or drain time. The results demonstrated a significant advantage in terms of renal functional preservation compared with DLS.^10^

Brassetti et al. compared sutureless and renorrhaphy techniques during purely off‐clamp robotic partial nephrectomy. The sutureless approach was shown to be safe and effective as renorrhaphy. They think that cortical renorrhaphy produces a wide wedge of ischaemic necrosis extending into the renal parenchyma.^19^

Based on these studies, the SLS technique appears to be feasible and safe in selected cases of MIPN. While in these studies, the tumours were rarely classified according to their growth depth. Due to nearing the larger vascular and collecting systems, LPN for renal tumours adjacent to the collecting system or renal sinus carries a higher risk of renal vessel injury than that for superficial tumours. Therefore, the overall quality of these evidences has been compromised.

We routinely performed SLS and DLS techniques in the LPN for several years, even for renorrhaphy of deep parenchymal defects. In this study, we selected cases with renal tumours in close proximity to the collecting system or renal sinus to compare the postoperative outcomes of SLS and DLS during LPN. To further minimize selection bias, cases with obviously opened collecting system or renal sinus during operation were excluded. For these cases, the openings were closed with 3‐0 barbed sutures followed by SLS or were closed by the inner‐layer renorrhaphy during DLS. The demographic and preoperative characteristics of the patients who underwent SLS were similar to those of the patients who underwent DLS. Both groups had similar intraoperative outcomes, except for the WIT. WIT was longer in the DLS group.

Compared with the DLS group, the SLS group showed no significant differences in the rates of overall complications and Clavien grade I–II complications. Clavien grade III–V complications were not observed. In addition, we did not find a significant difference in the decrease in postoperative haemoglobin levels and eGFR between the groups.

These results are consistent with those of previous studies, indicating that SLS does not significantly increase the incidence of postoperative bleeding or urinary leaks. This suggests that SLS may be safe and efficient during LPN surgery, even for deep renal defects, provided that no obviously opened collecting system or renal sinus is present. The reason for this result may be related to the following factors. After tumour resection, the venous openings among resection bed no longer have blood flow. Then, the openings of the renal arterial branch in resection bed are the most critical sites needing to be controlled for haemostasis. However, in most occasions, it is challenging to accurately locate the transected arterial openings when renal artery is clamped, which poses a major obstacle for precisely suture of the arterial bleeding points. Under these circumstances, every penetration of the inner‐layer basal sutures of DLS is blind, not precise. Therefore, whether using SLS (full‐thickness) technique or DLS technique, haemostasis of the renal defect primarily relies on the compression from cinching the defect closed. Thus, there is no significant difference in haemostatic effectiveness between the two methods.

The limitations of this study include its retrospective nature and small sample size. Although we performed propensity‐matched analysis to minimize potential confounders and selection biases, the selection bias cannot be completely ruled out. Future prospective randomized studies are needed to compare these two renal defect renorrhaphy techniques.

## 5 CONCLUSIONS

In our initial experience, the SLS technique during LPN for deep‐growing renal tumours is safe and feasible. Compared with the DLS technique, it has a shorter WIT without any added risk of postoperative complications. This technique is applicable to the renorrhaphy of deep renal defect in which collecting system and sinus opening are absent or limited.

## AUTHOR CONTRIBUTIONS

*Conceptualization*: Xiaofeng Xu. *Data curation*: Xiuquan Shi, Zhe Liu, Yuhao Chen and Jingping Ge. *Formal analysis*: Wen Cheng and Changjie Shi. *Funding acquisition*: Xiaofeng Xu. *Investigation*: Xiuquan Shi, Zhe Liu and Yuhao Chen. *Supervision*: Xuejun Shang. *Writing—original draft*: Xiumin Zhou. *Writing—review and editing*: Xiaofeng Xu. All authors read and approved the final version of the manuscript.

## CONFLICT OF INTEREST STATEMENT

The authors declare no competing interests.

## Data Availability

The data that support the findings of this study are available from the corresponding author upon reasonable request.
